# Volumetric changes in subcortical structures following repeated ketamine treatment in patients with major depressive disorder: a longitudinal analysis

**DOI:** 10.1038/s41398-020-00945-9

**Published:** 2020-08-03

**Authors:** Yan-Ling Zhou, Feng-Chun Wu, Wei-Jian Liu, Wei Zheng, Cheng-Yu Wang, Yan-Ni Zhan, Xiao-Feng Lan, Yu-Ping Ning

**Affiliations:** 1 The Affiliated Brain Hospital of Guangzhou Medical University (Guangzhou Huiai Hospital), Guangzhou, China; 2 Guangdong Engineering Technology Research Center for Translational Medicine of Mental Disorders, Guangzhou, China; 3 The First School of Clinical Medicine, Southern Medical University, Guangzhou, China

**Keywords:** Molecular neuroscience, Depression

## Abstract

Abnormal subcortical structures have been associated with major depressive disorder (MDD) and could be reversed by antidepressant treatment. To date no study has examined the relationship between subcortical volumes and repeated ketamine treatment. The current study investigated volume changes in specific subcortical structures and hippocampal subfields after six ketamine infusions. Forty-four patients with MDD received six subanesthetic dose infusions of ketamine. Depressive symptoms were assessed and magnetic resonance imaging scans were performed before and after six ketamine infusions. FreeSurfer software was used to process the T1 images and analyze the volumes of the subcortical regions and hippocampal subfields. After six ketamine infusions, increases were observed in the volumes of the left amygdala; the right hippocampus; the cornu ammonis 4 body, granule cell and molecular layer of the dentate gyrus body in the left hippocampus; and the cornu ammonis 4 head and molecular layer head in the right hippocampus. Positive correlations were found between symptom improvement and the pretreatment volumes of the right thalamus (*r* = 0.501; *P* = 0.001) and left subiculum head of the hippocampus (*r* = 0.471; *P* = 0.002), and changes in the volumes of the left amygdala (*r* = −0.452; *P* = 0.003) and the left cornu ammonis 4 body (*r* = −0.537; *P* < 0.001). Our findings provided evidence for critical roles of the amygdala and specific hippocampal subfields in the antidepressant effect of repeated ketamine treatment. Relatively larger volumes in right thalamus and left subiculum head in the hippocampus can predict a superior clinical outcome of ketamine treatment in MDD patients.

## Introduction

Major depression disorder (MDD) is a prevalent debilitating psychiatric illness that often has high rates of morbidity, mortality, and treatment failure taking traditional antidepressants^1^. All kinds of research progress of depression including antidepressant mechanisms, development of more effective therapeutic agents are very slowly due to poor understanding of neurobiology of depression. In recent years aberrant glutamatergic function-reduced glutamate metabolism, abnormal glutamate release, reduced post-synaptic glutamate receptors, and glutamate uptake deficits, is proposed to be related with depression^2^. These glutamatergic abnormalities are considered to induce excitatory neural toxicity, neuronal atrophy and synaptic loss, especially in subcortical, limbic, and cortical brain circuitries supporting control mood, emotion, and cognition^3^. Rodent studies have provided direct evidences of neuronal atrophy, loss, reduced synaptic density, and decreased neurogenesis in models of stress-induced depression^4,5^. Neuroimaging studies of depressed subjects show consistent and power evidences of atrophy of the prefrontal cortex, hippocampus, amygdala, and nucleus accumbens, and that is associated with depressive episodes, duration of illness and antidepressant treatment^6,7^.

The *N*-methyl-d-aspartate (NMDA) receptor antagonist ketamine has been shown to have a fast-acting antidepressant effect in several clinical trials^8–11^. The onset of antidepressant action occurs 40 min after a single intravenous infusion of ketamine (0.5 mg/kg over 40 min), and the peak effect occurs after 1 day^8,9,11^. Our previous studies found that patients received a thrice-weekly ketamine infusion regimen for 2 weeks sustained antidepressant response for longer^12^, similar with other studies^13,14^.

Ketamine rapidly reverses glutamatergic system and the neural structural plasticity hypothesis is one of the responsible mechanisms of its acute antidepressant action. In chronic unpredictable stress (CUS)-induced depression model rats, reduction of spine density, dendritic shrinkage, expression levels of synaptic proteins, and spine number were observed, and these structural deficits could be rapidly reversed by a single injection of ketamine^4,15^. Hippocampal atrophy was found in flinders sensitive line (FSL) rats, but a single dose of ketamine was able to increase volumes of the hippocampal cornu ammonis (CA)1 and granule cell layer (GCL) regions within 24 h of its infusion^16^. Clinical studies of depression have reported that neural structural plasticity within subcortical regions is associated with ketamine treatment^17–22^. Abdallah et al.^18^ tested the pretreatment hippocampal volume in 13 MDD patients who received a single infusion of ketamine and found a smaller left hippocampus volume was correlated with a greater symptom improvement 24 h after the infusion^18^. Niciu and colleagues reported that baseline subcortical volumes were not related to the antidepressant response in 55 unmedicated patients with treatment-resistant depression (TRD) within 24 h of a single infusion of ketamine^22^. However, combined with the brain-derived neurotrophic factor (BDNF) genotype, baseline bilateral thalamic volume was positively correlated with ketamine’s antidepressant response in met carriers. In another longitudinal study involving 16 MDD patients, Abdallah et al.^21^ found that a single infusion of ketamine reduced the left nucleus accumbens volume but increased the left hippocampal volume in patients who achieved remission following treatment. However, participants in previous studies received a single dose of ketamine and no studies have yet addressed the relationship between neural structural plasticity within subcortical regions and the response to repeated ketamine treatment.

Base on hypothesis about glutamatergic abnormalities in depression, we speculated that MDD patients would have abnormal subcortical volumes and that ketamine would induce the normalization of those subcortical volumes. Repeated ketamine treatment regimens administered for 2 to 3 weeks have been increasingly used to maintain the robust antidepressant effect for a longer duration relative to a single dose of infusion^12–14^. To date no study has examined whether the normalization of subcortical volumes in individuals received repeated infusions of ketamine is similar to them received a single infusion. Thus, we investigated the volumetric changes in the subcortical brain regions and hippocampal subfields in MDD subjects who received six infusions of ketamine. We hypothesized that repeated ketamine infusions would induce volume normalization of the subcortical brain regions.

## Materials and methods

### Participants

The data were obtained from a clinical trial (clinical trial number: ChiCTR-OOC-17012239) that examined the antidepressant effects of repeat ketamine treatment in depressed individuals^12,23,24^. This project started in September 2016 and is ongoing. The sample size of this project should be 92 to ensure a power of 90% and an α of 0.05 by using PASS software (NCSS, USA). According to the previous longitudinal study that 16 MDD patients were included and reported the left nucleus accumbens volume reduced and the left hippocampal volume increased after a single dose of ketamine treatment^21^, 44 patients who completed six infusions of ketamine and underwent magnetic resonance imaging (MRI) scanning before and after treatment were included in the present study.

The protocol was approved by the Clinical Research Ethics Committee of the Affiliated Brain Hospital of Guangzhou Medical University. All participants were recruited from the Affiliated Brain Hospital of Guangzhou Medical University and provided informed consent.

MDD subjects were diagnosed by two experienced psychiatrists using a structured clinical interview for Diagnostic and Statistical Manual of Mental Disorders-5 (DSM-5, SCID). The inclusion criteria for MDD patients were as follows: aged 18–65 years; had a 17-item Hamilton Depression Rating Scale (HAMD-17) score ≥17 at screening; had no psychotic symptoms; had experienced treatment resistance defined as the failure of two adequate antidepressant trials or a suicidal tendency confirmed by a Beck Scale for Suicide Ideation-part I score ≥2 at screening; be free of comorbid alcohol or substance abuse or dependence; without the presence of any serious or unstable medical conditions; and had no MRI contraindications. Forty-five HCs were recruited from the community; they met the same exclusion criteria but had no personal or family history of psychiatric illness based on the SCID, was not taking any medications and had no MRI contraindications.

### Study design

MDD subjects received a thrice-weekly ketamine infusion regimen for two weeks. The detailed methods have been described in our previous studies^12,23,24^. Following an overnight fast, patients received a subanesthetic dose (0.5 mg/kg) of ketamine diluted in saline administered over 40 min via IV intravenous pump continuous infusion. The hemodynamic and clinical status of patients was monitored during the injection. If a patient was taking a psychiatric medication at screening, a stable dose for ≥4 weeks had to have been achieved before the ketamine infusion, and these patients were maintained on their stable doses of medications throughout the infusion period.

MDD subjects were assessed depression symptom and underwent MRI scans 1 day before ketamine infusion and again at 1 day after the sixth ketamine infusion (pretreatment and posttreatment). The HCs were only scanned once.

### Rating scales

The depression symptom was assessed via the Montgomery-Asberg Scale (MADRS). The ketamine’s antidepressant effect was calculated by the average change in the MADRS scores (pretreatment minus posttreatment). A response to ketamine treatment was defined as a ≥50% improvement in the MADRS scores posttreatment^25^. The MADRS was administered by psychiatrists with abundant clinical experience, with an interclass correlation coefficient (ICC) >0.90.

### MRI and data processing

Brain MRIs were acquired with a 3.0 T MRI scanner (Achieva X-series, Philips Medical Systems, Best, the Netherlands) using a circular polarized birdcage head coil. Structural T1-weighted images were acquired in a sagittal orientation employing the spoiled gradient recall (SPGR) sequence with the following parameters: slice thickness = 1 mm; voxel size = 1 × 1 × 1 mm^3^; and number of slices = 188. The total acquisition time was 652.9 s. The subjects were told to hold still in the scanner with their eyes closed during the scan.

Image segmentation and the estimation of the subcortical volumes were performed using the publicly available FreeSurfer 6.0 software with the standard parameters (Harvard Medical School, Boston, USA; http://surfer.nmr.mgh.harvard.edu/). This automated processing tool performs the isolation and removal of nonbrain tissue (i.e., “skullstripping”), subcortical segmentation, and estimation of the total intracranial volume (TIV), whole brain volume and subcortical brain region volume^26,27^. All segmented images were reviewed by an imaging expert to rule out the presence of errors or misclassifications for postprocessing quality control. This imaging expert was blinded to the group classification of each subject. One patient’s posttreatment image data was of poor quality; to salvage this poor segmentation, we performed a manual correction, but it was still unsatisfactory. Therefore, these data were discarded, and the data from 44 subjects with MDD were included in the final analyses. According to the technical details described in previous studies, the automated segmentation of the subcortical volumes and assignment of a neuroanatomic label to each voxel on MRI were performed, and the volume was determined based on probabilistic information automatically estimated from a manually labeled training set. In this study, we finally obtained the TIV and the volumes of six specific brain regions, namely, the thalamus, caudate, putamen, pallidum, hippocampus, and amygdala.

Next, the segmentation of the hippocampal subfields was performed using a novel automated algorithm that was included in FreeSurfer^28^. The hippocampal subfield atlas was derived from high resolution (0.13 mm) ex vivo MRI data of postmortem medial temporal tissue obtained with a 7-T scanner. This new automatic algorithm was able to accurately identify the GCL within the dentate gyrus, the molecular layer (ML) within the subiculum and the CA subfields, as well as the hippocampal tail^29^. We focused on the volumes of 19 hippocampal subfields: CA1 (head and body), CA3 (head and body), CA4 (head and body), fimbria, granule cell and molecular layer of the dentate gyrus (GC-ML-DG, head and body), hippocampal–amygdaloid transition area (HATA), fissure, tail, ML (head and body), parasubiculum (Para), presubiculum (Pre, head and body), and subiculum (Sub, head and body).

### Statistical analysis

First, baseline demographics variables were compared between the groups (MDD and HCs, ketamine responders and nonresponders) using Student’s *t* tests for continuous variables and chi-squared tests for categorical variables.

Next, the group comparisons of imaging volumes between MDD and HCs were performed with multivariate analysis of covariance (MANCOVA), with age, sex, body mass index (BMI), and TIV as covariates. The diagnosis group (MDD and HCs) was the independent variable, while the subcortical volumes and the hippocampal subfield volumes were the dependent variables. Post hoc analysis was also performed among HCs, ketamine responders and nonresponders for each subcortical brain region and hippocampal subfield. An effect size was calculated to measure their difference.

Then, MANCOVAs were separately performed for the subcortical structures and subfields of the hippocampus, with age, sex, BMI, combined use of mood stabilizer/benzodiazepine/antipsychotic, and TIV as covariates. Post hoc paired *t*-test was performed for each subcortical brain region and hippocampal subfield using the pretreatment and posttreatment volumes of whole MDD subjects, as well as the subgroups of responders and nonresponders. An effect size was calculated to measure their change before and after ketamine treatment.

Finally, to examine the associations between symptom improvement and the volumes of the subcortical regions and hippocampal subfields, partial correlation analyses were performed with the reduction in MADRS scores and the baseline volumes of the subcortical regions and hippocampal subfields and their changes after ketamine treatment. The control variables were age, sex, BMI, TIV and baseline MADRS scores.

The statistical analyses were performed with SPSS 22. The Bonferroni correction was used to counteract the problem of multiple comparisons with the number of regions (6 for subcortical regions, 19 for subfields of the hippocampus). *P*-values <0.0083 (0.05/6) were considered significant for the subcortical volumes, and *P*-values <0.0026 (0.05/19) were considered significant for the hippocampal subfield volumes. The raw *P*-values are reported in the Results section. All reported *P*-values are two sided.

## Results

### Demographics

Forty-four patients who completed six infusions of ketamine, with normal anatomical MRI scans for subcortial volume before and after ketamine treatment, were included in the present study. The characteristics of the patients and the HCs are shown in Table 1. The MDD and HC groups had no significant difference in age, sex or BMI (all *P* > 0.05).

**Table 1:** Characteristics of patients with major depressive disorder and healthy controls.

Variables	MDD (*N* = 44)	RES (*N* = 27)	NRES (*N* = 17)	HCs (*N* = 45)	MDD vs HCs		RES vs NRES	
	*N* (%)	*N* (%)	*N* (%)	*N* (%)	*χ*^2^	*P*	*χ*^2^	*P*
Male	16 (36.3)	9 (33.3)	7 (41.2)	21 (46.7)	1.147	0.284	0.277	0.598
Treatment resistance	36 (81.8)	23 (85.2)	13 (76.5)	–	–	–	0.108	0.743
With suicidality	27 (61.4)	16 (59.3)	11 (64.7)	–	–	–	0.131	0.718
Recurrent	28 (63.6)	16 (59.3)	12 (70.6)	–	–	–	0.579	0.447
Depression subtypes	–	–	–	–	–	–	1.148^d^	0.764
Melancholic	8 (18.2)	6 (22.2)	2 (11.8)	–	–	–	–	–
Anxious	13 (29.5)	8 (29.6)	5 (29.4)	–	–	–	–	–
Melancholic-anxious	15 (34.1)	9 (33.3)	6 (35.3)	–	–	–	–	–
No subtype	8 (18.2)	4 (14.8)	4 (23.5)	–	–	–	–	–
Current pharmacotherapies								
≥2 antidepressants	10 (22.7)	7 (25.9)	3 (17.6)	–	–	–	0.417^d^	0.716
Mood stabilizer	6 (13.6)	5 (18.5)	1 (5.9)	–	–	–	1.570^d^	0.380
Benzodiazepine	24 (54.5)	14 (51.9)	9 (52.9)	–	–	–	0.005	1.000
Antipsychotic	28 (63.6)	15 (55.6)	12 (70.6)	–	–	–	1.011	0.360

	Mean (SD)	Mean (SD)	Mean (SD)	Mean (SD)	*t*	*P*	*t*	*P*
Age	35.2 (12.2)	39.0 (11.7)	29.9 (11.2)	33.0 (11.2)	−0.907	0.367	−2.541	0.015
Education (years)	12.0 (3.2)	11.8 (3.2)	12.1 (3.4)	12.0 (2.9)	0.034	0.973	0.301	0.765
BMI (kg/m^2^)	22.8(3.3)	24.0(2.7)	21.2(3.2)	22.1(2.8)	−1.024	0.309	−3.087	0.004
Duration of illness (months)	76.6(77.3)	86.5(77.3)	64.4(78.5)	–	–	–	−0.919	0.363
Dose of antidepressant (mg/day)^a^	44.5(23.1)	46.4(22.9)	42.2(24.4)	–	–	–	−0.576	0.568
Dose of antipsychotic (mg/day)^b^	183.8(109.4)	199.1(87.3)	170.0(126.8)	–	–	–	−0.814	0.421
Dose of benzodiazepine (mg/day)^c^	2.9(1.2)	2.9(1.2)	2.9(1.4)	–	–	–	−0.155	0.878
Baseline MADRS score	32.0(8.6)	32.0(8.6)	32.4(9.1)	–	–	–	0.130	0.897

*HCs* healthy controls, *MDD* major depressive disorder, *RES* responders, *NRES* non-responders, *MADRS* Montgomery-Asberg Scale.

^a^Fluoxetine equivalent dose.

^b^Chloropromazine equivalent dose.

^c^Lorazepam equivalent dose.

^d^Fisher’s exact test.

### Efficacy of ketamine treatment

The average change in MADRS scores after six infusions of ketamine was significant (pretreatment MADRS minus posttreatment MADRS = 17.0±10.2; *t* = −9.208, *P* < 0.001, Cohen’s *d* = −1.791). Twenty-seven patients (61.4%) showed significant improvement (more than a 50% decrease in MADRS scores) after receiving six infusions of ketamine. No significant differences were observed in age, sex, BMI, and baseline MADRS scores between ketamine responders and nonresponders (all *P* >0.05, Table 1).

### Volume differences at baseline

**Fig. 1:**
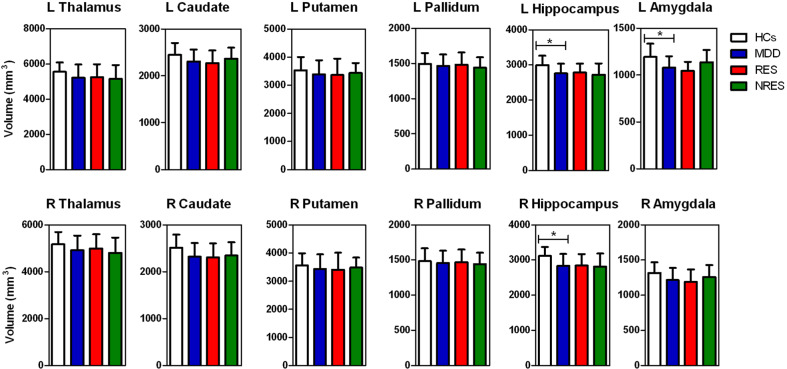
Baseline subcortical volumes of responders and non-responders of ketamine and healthy controls. MDD had smaller volumes than HCs in the left amygdala and the bilateral hippocampus. HCs healthy controls, MDD major depressive disorder, RES responders, NRES non-responders, L left, R right. *P-value significant after the Bonferroni correction.

Smaller volumes were observed in the bilateral thalamus, bilateral hippocampus and bilateral amygdala of the MDD group compared with the volumes in the HC group at baseline, while only the left amygdala (*F* = 16.403, *P* < 0.001, Cohen’s *d* = −0.886) and bilateral hippocampus (left *F* = 11.926, *P* = 0.001, Cohen’s *d* = −0.829; right *F* = 15.719, *P* < 0.001, Cohen’s *d* = −0.948) were significant after Bonferroni correction. There was no significant volume difference in any of the subcortical regions between responders and nonresponders (Fig. 1 and Supplementary Table 1).

**Fig. 2:**
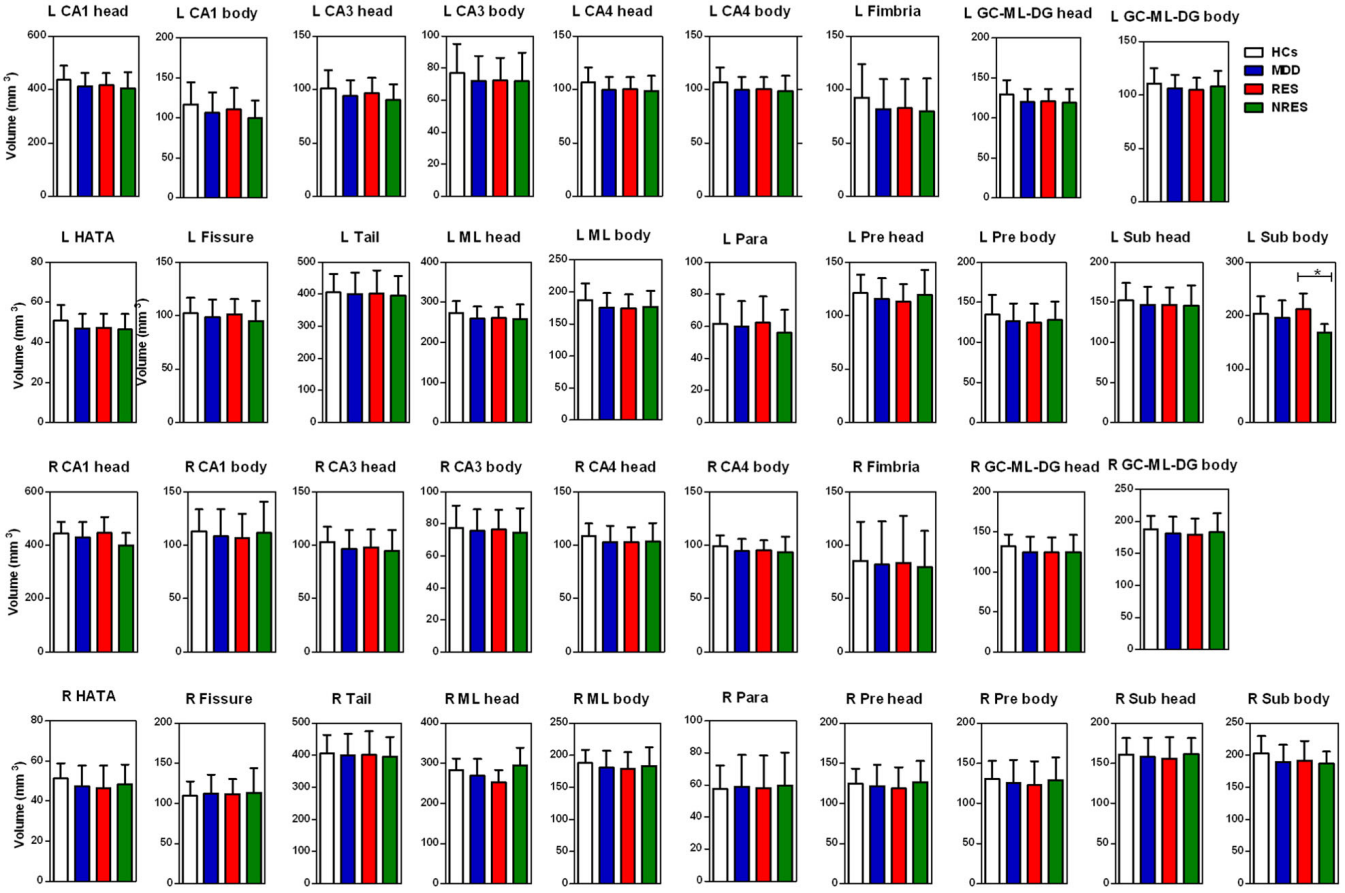
Baseline hippocampal subfield volumes of responders and non-responders of ketamine and healthy controls. Responders had larger volumes than nonresponders in the left Sub body. HCs healthy controls, MDD major depressive disorder, RES responders, NRES non-responders, L left, R right, GC granule cell, ML molecular layer, CA cornu ammonis, DG dentate gyrus, HATA hippocampal-amygdaloid transition area, Para parasubiculum, Pre presubiculum, Sub subiculum. *P-value significant after the Bonferroni correction.

Significant differences in baseline volumes between MDD patients and HCs were observed in the following left hippocampal subfields: CA1 body, CA1 head, CA4 head, GC-ML-DG head, HATA, ML head, and ML body; in addition significant differences were observed in the following right hippocampal subfields: GC-ML-DG body and Sub body. However, no significant differences in the volumes of the subfields were found after Bonferroni correction. Post hoc analysis showed that responders had larger volumes than nonresponders in the left Sub body and the right ML head, while only the left Sub body (*F* = −3.840, *P* = 0.001, Cohen’s *d* = 1.861) retained significance after Bonferroni correction (Fig. 2 and Supplementary Table 2).

### Effects of ketamine treatment on subcortical regions and the hippocampal subfields

**Fig. 3:**
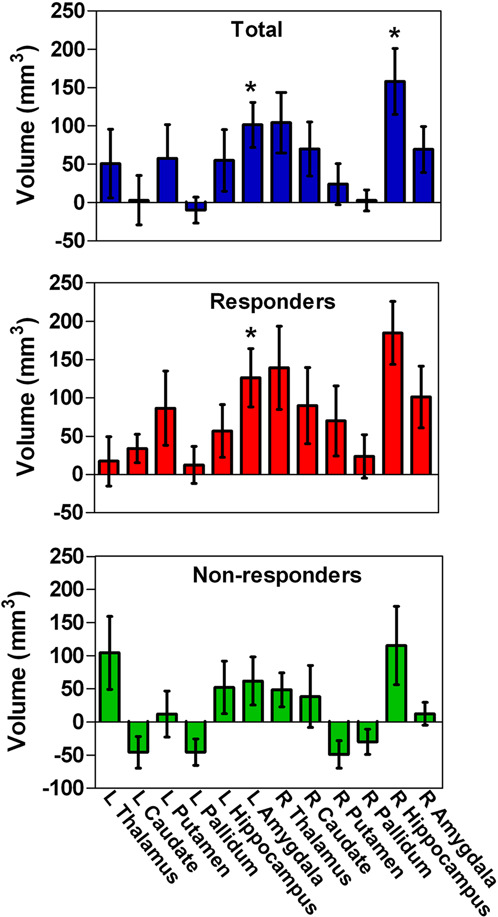
The volume increase of subcortical regions in responders and non-responders of ketamine. Responders showed significant increase in volumes of the left amygdala. L left, R right. *P-value significant after the Bonferroni correction.

Ketamine had no significant effect on subcortical volumes according to MANCOVA (Wilks’ Lambda = 0.565, *F* = 2.052, *P* = 0.052). In the post hoc analysis, significant increases in volume were found in the left amygdala, right thalamus, hippocampus, and amygdala (all uncorrected *P* < 0.05), while only the left amygdala (*t* = 3.438, *P* = 0.001, Cohen’s *d* = 0.640) and right hippocampus (*t* = 2.983, *P* = 0.005, Cohen’s *d* = 0.485) were significant after Bonferroni correction. Post hoc analysis showed a reduction in left pallidum volumes in the nonresponders and an increase in the bilateral amygdala and right hippocampal volumes in the responders following treatment (all uncorrected *P* < 0.05), while only the left amygdala (*t* = 3.310, *P* = 0.003, Cohen’s *d* = 0.809) of the responders was significant after Bonferroni correction (Fig. 3 and Supplementary Table 3).

**Fig. 4:**
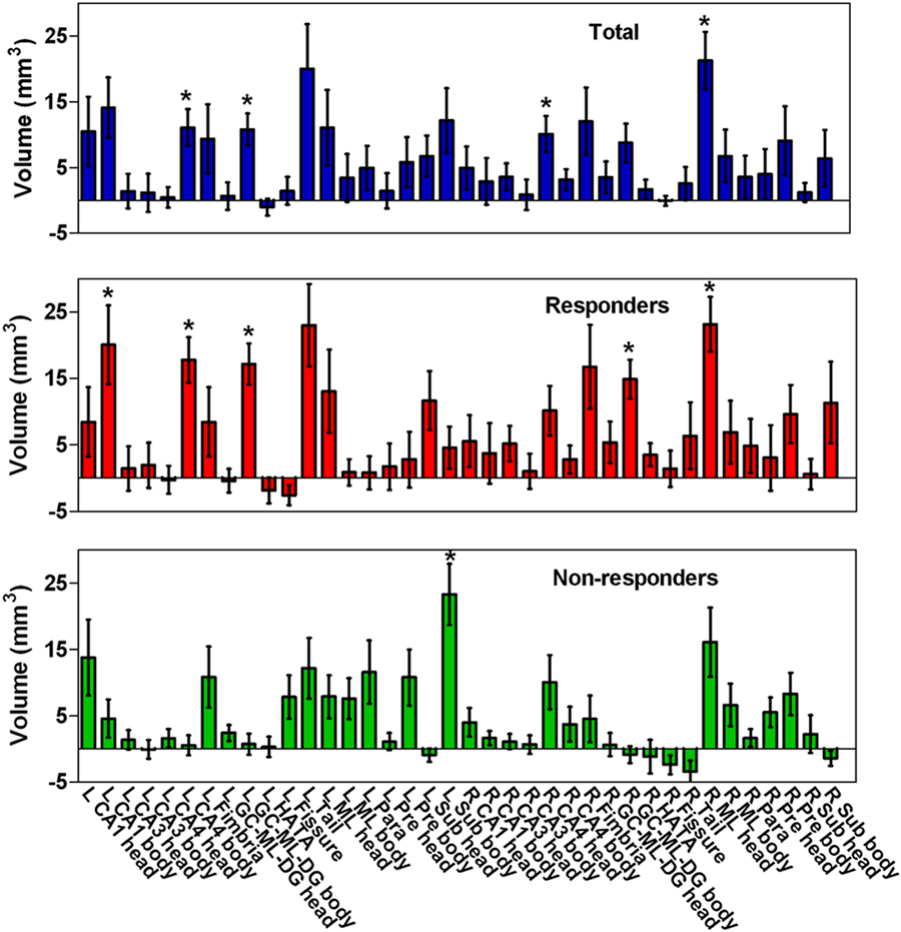
The volume increase of hippocampal subfields in responders and non-responders of ketamine. Responders showed significant increase in volumes of the left CA1 body, left CA4 body, left GC-ML-DG body, right GC-ML-DG body, and right ML head; nonresponders showed significant increase in volumes of the left Sub body. L left, R right, GC granule cell, ML molecular layer, CA cornu ammonis, DG dentate gyrus, HATA hippocampal-amygdaloid transition area, Para parasubiculum, Pre presubiculum, Sub subiculum. *P-value significant after the Bonferroni correction.

With regard to the hippocampal subfields, a significant effect of ketamine on volume was found according to MANCOVA (Wilks’ Lambda = 0.020, *F* = 7.792, *P* = 0.008). Among the subfields of the left hippocampus, increased volumes following ketamine treatment were observed in the CA1 body, CA4 body, GC-ML-DG body, Sub head and Sub body, and increased volumes of the CA4 head, GC-ML-DG body, and ML head were observed in the right hippocampus (all uncorrected *P* < 0.05); however, only the left CA4 body (*t* = 3.989, *P* < 0.001, Cohen’s *d* = 0.806), left GC-ML-DG body (*t* = 4.401, *P* < 0.001, Cohen’s *d* = 0.822), right CA4 head (*t* = 3.677, *P* = 0.001, Cohen’s *d* = 0.636) and right ML head (*t* = 3.500, *P* = 0.001, Cohen’s *d* = 0.596) remained significant after Bonferroni correction. Post hoc analysis found significant increases in volumes in the left CA1 body (*t* = 3.388, *P* = 0.002, Cohen’s *d* = 0.921), left CA4 body (*t* = 5.201, *P* < 0.001, Cohen’s *d* = 1.444), left GC-ML-DG body (*t* = 5.518, *P* < 0.001, Cohen’s *d* = 1.413), right GC-ML-DG body (*t* = 5.066, *P* < 0.001, Cohen’s *d* = 1.249), and right ML head (*t* = 3.700, *P* < 0.001, Cohen’s *d* = 0.888) of the responders and in the left Sub body (*t* = 5.263, *P* < 0.001, Cohen’s *d* = 1.295) of the nonresponders. In addition, all these results remained significant after Bonferroni correction (Fig. 4 and Supplementary Table 4).

### Relation to clinical change

After Bonferroni correction for multiple comparisons, larger pretreatment volumes in the right thalamus (*r* = 0.501, *P* = 0.001) and left Sub head hippocampal subfield (*r* = 0.471, *P* = 0.002) were correlated with a greater reduction in MADRS scores. Changes in the volumes of the left amygdala (*r* = −0.452, *P* = 0.003) and the left CA4 body (*r* = −0.537, *P* < 0.001) were negatively correlated with reductions in MADRS scores following ketamine (Supplementary Tables 5, 6).

## Discussion

The current study showed that there was an increase in the volumes of the right hippocampus and left amygdala after six infusions of ketamine in patients with MDD. Another important finding was that patients with larger volumes of the right thalamus and the subiculum head of the left hippocampus pretreatment are more likely to benefit from repeated ketamine infusions.

The reduction in bilateral hippocampal volumes in MDD patients replicated most of preclinical and clinical findings of depression-related atrophy in the hippocampus^5–7^. Following six infusions of ketamine, increases in the volumes of the right hippocampal region, left CA4 body, left GC-ML-DG body, right CA4 head, and right ML head were observed. Trend effects were observed in the left CA1 body, left Sub head and body, and right GC-ML-DG body. The present findings were in line with those of a clinical study in which increased hippocampal volumes were observed in patients who achieved remission following an infusion of ketamine, yet the study reported that the increased volumes were localized to the left side^18^. Animal studies also found that a single ketamine injection could induce increased volumes of the hippocampal CA1 and GCL regions twenty-four hours after the treatment in FSL rats^16^, and reverse the reduction of the number of granule cells in FSL-treated rats^30^. These findings support that rapid hippocampal structural plasticity induced by ketamine may be a potential mechanism underlying its fast antidepressant action. Similarly, other treatments for depression, such as treatment with antidepressant medications and electroconvulsive therapy, also can enhance hippocampal structural plasticity^31,32^. Furthermore, related results were also reported in a study utilizing functional MRI and showing a reduced functional connectivity of the amygdalo-hippocampus with regard to emotional stimulation following ketamine injection^20^.

The amygdala plays a crucial role in emotional behavior, the stress response and vulnerability to depression. Structural changes in the amygdala have been associated with chronic stress and early adversity^33,34^. Morphological changes in the amygdala in patients with MDD have been reported in many studies, but the findings are inconsistent and may depend on the different illness phases, duration of illness, recurrence, family history, and antidepressant treatment of MDD^35–37^. For instance, amygdala volumes were decreased in individuals with recurrent MDD and a family history of MDD, while they were enlarged in a current first episode of MDD and in patients receiving antidepressant medications^35–37^. Our findings showed that a smaller amygdala volume in individuals with MDD compared to the volume in HCs could be reversed by six ketamine infusions. Accordingly, an association between enlargement in the volumes of the left amygdala and clinical improvement was found in our study. The increase in the volume of the amygdala following ketamine treatment might be attributable to the neural structural plasticity induced by ketamine, similar to that in the hippocampus. However, there was not enough evidence to support this hypothesis, although the adult amygdala has been described as undergoing neurogenesis based on evidence of new neurons in the basolateral amygdala in brain injury models and in the medial and central nuclei of the amygdala in voles under normal physiological conditions and undergoing hormonal treatments^38–40^.

Although ketamine is considered the most powerful antidepressant medication for patients with treatment-resistant depression, more than 30% of patients with TRD do not adequately respond to ketamine^11,41^, even with serial infusions^12–14^. Thus, it is vitally important to identify the predictors of a response to repeated infusions of ketamine. In the exploratory analysis in our sample, baseline volumes of the right thalamus and left Sub head in the hippocampus were positively correlated with changes in MADRS scores, such that patients with relatively larger right thalamus and left Sub head volumes had greater improvements in depressive symptoms following six doses of ketamine. These findings disagreed with the findings of previous studies that showed that patients with smaller pretreatment hippocampal volumes were more likely to achieve better clinical improvements and that smaller left and right thalamic volumes were correlated with the antidepressant response to ketamine in BDNF 66Met allele carriers^18,22^. However, the present findings were in line with those of our previous study that showed that patients with better neurocognitive function, an index that could reflect hippocampal function, may be more likely to benefit from ketamine treatment^24^. Moreover, a similar correlation was observed in patients receiving traditional antidepressants: larger pretreatment hippocampal volumes predicted a better antidepressant response and recovery^42^. One potential explanation for this is that patients with larger hippocampal volumes have a higher intrinsic “restorative” capacity of neuronal plasticity; thus, they could tend to achieve more synergistic effects with the antidepressant treatment^42^. Also note that the present study sample was relatively young, with a short duration of illness, mixed (TRD and suicidal patients) on a high frequency of antipsychotic medicines (63.6%) as compared to other reports of TRD with ketamine. Different object the study chooses may result in the different conclusion that was at odds with previous neuroimaging result.

The potential limitations of this study should be considered. First, the depressive subjects included in this study lacked a washout period and continued receiving their previous medications during ketamine treatment, and these medications can produce changes in regional brain volumes. For example lithium and valproate, as well as antidepressants have been reported to increase neurogenesis and hippocampal brain volume. Therefore, the findings cannot be generalized to medication-free patients. This limitation was imposed by ethical considerations that make the discontinuation of medication difficult to carry out. In fact, combination therapies with ketamine and other antidepressants are being increasingly used in clinical trials in larger “real-world” patient cohorts. However, this effect could be attenuated by the requirement for stable medication doses maintained for 4 weeks before ketamine infusion, and ongoing medication was included as a covariate in the statistical analyses, increasing the stability of the results. Second, the depressed subjects included in this study were complicated by treatment resistance, suicidality or both. The sample size of the MDD group limited a full investigation of the impacts of these complications; further study in a more homogeneous population is needed. Third, there was no measure of childhood maltreatment, a major determinant of hippocampal and other subcortical region size.

In conclusion, our present study showed that six ketamine infusions may induce volume increases in left amygdala, right hippocampus and the following specific hippocampal subfields: the left CA4 body, left GC-ML-DG body, right CA4 head, and right ML head. We also found that patients with relatively larger right thalamus and left subiculum head volumes may have better symptom improvement following six doses of ketamine. The volume normalization of the subcortical brain regions supports a modification of the potential for neural structural plasticity by ketamine and contributes to our understanding of its mechanism of antidepressant action. Using these brain regions as biomarkers may improve our ability to choose the best treatment strategies for depressed patients who are experiencing treatment resistance or suicidality.

## Supplementary Information

Supplementary Information
