# Supplementary material for: Volumetric changes in subcortical structures following repeated ketamine treatment in patients with major depressive disorder: a longitudinal analysis
Source: Transl Psychiatry. 2020 Aug 3;10:264. doi: 10.1038/s41398-020-00945-9 (PMC7400625; doi:10.1038/s41398-020-00945-9)
Supplement: Supplementary file 1 — Supplementary Information [file 41398_2020_945_MOESM1_ESM.doc]

**Supplementary table 1. Volume Differences in the subcortical regions among responders, non-responders, and healthy controls.**

| Subcortical regions | MDD | RES | NRES | HCs | MDD VS HCs | | RES vs NRES | |
| --- | --- | --- | --- | --- | --- | --- | --- | --- |
|  | Mean(SD) | Mean(SD) | Mean(SD) | Mean(SD) | F | *P* | t | *P* |
| **Left** |  |  |  |  |  |  |  |  |
| Thalamus | 5216.7(750.6) | 5249.9(743.1) | 5163.9(782.3) | 5564.3(520.8) | 5.021 | 0.028 | 0.709 | 1.000 |
| Caudate | 2308.3(254.4) | 2273.5(265.4) | 2363.5(232.8) | 2445.2(257.0) | 0.026 | 0.873 | -0.194 | 1.000 |
| Putamen | 3388.1(498.8) | 3361.3(576.8) | 3430.5(353.2) | 3535.6(460.3) | 1.492 | 0.225 | -0.073 | 1.000 |
| Pallidum | 1466.7(163.2) | 1482.4(173.0) | 1441.8(147.8) | 1495.2(153.7) | 0.537 | 0.466 | 0.586 | 1.000 |
| Hippocampus | 2762.9(278.0) | 2790.1(247.1) | 2719.6(324.3) | 2992.7(276.4) | 11.926 | 0.001* | 0.230 | 1.000 |
| Amygdala | 1080.8(118.0) | 1046.0(95.5) | 1136.2(131.5) | 1194.9(138.5) | 16.403 | <0.001* | -2.128 | 0.109 |
| **Right** |  |  |  |  |  |  |  |  |
| Thalamus | 4912.9(629.7) | 4985.9(609.9) | 4796.9(661.5) | 5172.3(521.1) | 3.990 | 0.049 | 1.295 | 0.597 |
| Caudate | 2323.0(288.9) | 2308.3(295.2) | 2346.5(286.0) | 2511.6(277.0) | 1.654 | 0.202 | 0.331 | 1.000 |
| Putamen | 3436.0(514.1) | 3408.1(597.3) | 3480.4(356.6) | 3550.4(437.1) | 0.576 | 0.450 | -0.036 | 1.000 |
| Pallidum | 1457.8(173.5) | 1467.5(182.2) | 1442.4(162.9) | 1483.7(182.9) | 0.503 | 0.480 | -0.148 | 1.000 |
| Hippocampus | 2835.7(335.5) | 2846.2(322.9) | 2819.1(364.1) | 3117.3(253.8) | 15.719 | <0.001* | 0.301 | 1.000 |
| Amygdala | 1216.6(173.3) | 1189.8(174.8) | 1259.0(167.3) | 1318.1(151.0) | 6.817 | 0.011 | -1.382 | 0.512 |

HCs, healthy controls; MDD, major depressive disorder; RES, responders; NRES, non-responders.

**P*-value significant after the Bonferroni correction.

**Supplementary table 2. Volume Differences in the hippocampal subfields among responders, non-responders, and healthy control subjects.**

| Subfields | MDD | RES | NRES | HCs | MDD VS HCs | | RES vs NRES | |
| --- | --- | --- | --- | --- | --- | --- | --- | --- |
|  | Mean(SD) | Mean(SD) | Mean(SD) | Mean(SD) | F | *P* | t | *P* |
| **Left** |  |  |  |  |  |  |  |  |
| CA1 head | 411.4(50.6) | 415.4(45.3) | 405.2(59.0) | 435.2(52.4) | 4.244 | 0.043 | 0.761 | 1.000 |
| CA1 body | 106.3(25.5) | 110.4(27.0) | 99.8(22.2) | 117.1(27.5) | 4.597 | 0.035 | -3.581 | 0.062 |
| CA3 head | 93.8(14.7) | 96.3(14.3) | 89.9(14.9) | 100.6(17.5) | 3.903 | 0.052 | 1.165 | 0.742 |
| CA3 body | 712.0(15.6) | 72.2(14.1) | 71.6(18.2) | 76.8(18.2) | 1.634 | 0.205 | 0.044 | 1.000 |
| CA4 head | 99.4(12.4) | 99.9(11.6) | 98.6(13.9) | 106.4(14.2) | 5.734 | 0.019 | 0.319 | 1.000 |
| CA4 body | 94.4(10.8) | 93.8(9.6) | 95.4(12.7) | 98.2(13.0) | 1.901 | 0.172 | -0.724 | 1.000 |
| Fimbria | 81.4(28.0) | 82.5(27.0) | 79.6(30.3) | 92.4(31.2) | 3.184 | 0.078 | 0.084 | 1.000 |
| GC-ML-DG head | 120.3(15.6) | 121.3(14.7) | 118.8(17.3) | 129.3(17.9) | 5.939 | 0.017 | 0.574 | 1.000 |
| GC-ML-DG body | 105.9(12.6) | 104.6(11.2) | 107.9(14.8) | 110.5(14.4) | 2.085 | 0.153 | -0.986 | 0.981 |
| HATA | 47.0(7.2) | 47.3(7.2) | 46.5(7.6) | 50.8(7.9) | 5.887 | 0.017 | -0.166 | 1.000 |
| Fissure | 98.7(16.4) | 101.0(14.5) | 94.9(18.8) | 102.3(14.3) | 1.150 | 0.287 | 1.159 | 0.749 |
| Tail | 392.2(63.1) | 400.2(56.1) | 379.4(73.0) | 413.3(56.4) | 2.509 | 0.117 | 0.864 | 1.000 |
| ML head | 259.4(30.5) | 260.6(26.6) | 257.4(36.7) | 273.6(29.3) | 4.421 | 0.039 | 0.558 | 1.000 |
| ML body | 175.2(22.7) | 174.5(21.5) | 176.4(25.0) | 187.1(26.0) | 4.563 | 0.036 | -0.285 | 1.000 |
| Para | 59.8(15.7) | 62.2(16.4) | 56.0(14.2) | 61.6(18.4) | 0.244 | 0.623 | 1.191 | 0.711 |
| Pre head | 115.7(19.2) | 113.2(16.2) | 119.6(23.1) | 120.9(17.4) | 1.224 | 0.272 | -0.980 | 0.991 |
| Pre body | 126.2(22.5) | 124.8(22.9) | 128.4(22.3) | 134.6(24.7) | 2.029 | 0.158 | -0.304 | 1.000 |
| Sub head | 146.5(22.6) | 146.9(21.3) | 146.1(25.2) | 152.2(21.9) | 1.098 | 0.298 | 0.044 | 1.000 |
| Sub body | 195.3(32.7) | 212.1(29.3) | 168.6(15.3) | 204.2(32.6) | 3.447 | 0.067 | 3.840 | 0.001* |
| **Right** |  |  |  |  |  |  |  |  |
| CA1 head | 428.1(58.8) | 445.5(60.4) | 400.5(45.3) | 443.3(44.7) | 1.704 | 0.195 | 3.016 | 0.010 |
| CA1 body | 108.7(25.1) | 106.7(22.6) | 111.8(29.1) | 113.0(20.6) | 0.403 | 0.527 | -0.507 | 1.000 |
| CA3 head | 96.7(17.6) | 97.9(17.0) | 94.9(19.0) | 103.1(14.1) | 2.642 | 0.108 | 1.260 | 0.634 |
| CA3 body | 75.7(13.3) | 76.5(12.2) | 74.5(15.2) | 77.3(13.9) | 0.265 | 0.608 | 0.244 | 1.000 |
| CA4 head | 103.2(15.0) | 103.1(13.8) | 103.4(17.1) | 108.9(11.5) | 2.959 | 0.089 | 0.384 | 1.000 |
| CA4 body | 94.5(11.5) | 95.0(9.6) | 93.8(14.4) | 99.0(10.5) | 3.246 | 0.075 | 0.213 | 1.000 |
| Fimbria | 82.2(40.0) | 83.8(43.9) | 79.6(34.0) | 85.6(36.5) | 0.280 | 0.598 | 0.199 | 1.000 |
| GC-ML-DG head | 124.8(19.4) | 124.8(18.5) | 124.9(21.4) | 132.1(14.7) | 2.890 | 0.093 | 0.480 | 1.000 |
| GC-ML-DG body | 105.7(12.9) | 105.5(10.8) | 106.0(16.0) | 111.6(12.1) | 4.170 | 0.044 | -0.203 | 1.000 |
| HATA | 47.3(10.5) | 46.5(11.1) | 48.5(9.7) | 51.4(7.5) | 3.451 | 0.067 | -0.418 | 1.000 |
| Fissure | 112.3(23.9) | 111.6(19.2) | 113.3(30.6) | 109.8(17.9) | 0.205 | 0.652 | -0.975 | 0.998 |
| Tail | 399.9(67.8) | 402.2(72.6) | 396.4(61.3) | 405.5(57.7) | 0.047 | 0.829 | 0.087 | 1.000 |
| ML head | 269.2(41.6) | 252.9(30.9) | 295.1(44.1) | 283.7(28.4) | 1.802 | 0.183 | -3.372 | 0.003 |
| ML body | 181.0(26.4) | 179.5(25.1) | 183.5(28.9) | 188.2(20.7) | 1.403 | 0.240 | -0.507 | 1.000 |
| Para | 58.8(20.01 | 58.1(20.3) | 59.8(20.4) | 57.7(14.6) | 0.427 | 0.515 | 0.020 | 1.000 |
| Pre head | 122.0(26.2) | 119.0(26.2) | 126.7(26.3) | 124.5(19.2) | 0.064 | 0.801 | -0.985 | 0.983 |
| Pre body | 125.4(29.0) | 123.0(29.9) | 129.1(28.1) | 131.0(22.3) | 0.506 | 0.479 | -0.756 | 1.000 |
| Sub head | 158.0(24.1) | 155.8(26.6) | 161.6(19.7) | 161.3(20.2) | 0.355 | 0.553 | -1.205 | 0.695 |
| Sub body | 190.1(26.5) | 192.1(30.3) | 186.9(19.3) | 203.0(27.2) | 5.806 | 0.018 | -0.019 | 1.000 |

HCs, healthy controls; MDD, major depressive disorder; RES, responders; NRES, non-responders. GCL, granule cell layer; ML, molecular layer; CA, cornu ammonis; DG, dentate gyrus; HATA, hippocampal-amygdaloid transition area; Para, parasubiculum; Pre, presubiculum; Sub, subiculum.

**P*-value significant after the Bonferroni correction.

**Supplementary table 3. Results of a post-hoc paired t-test for each subcortical brain region between before and after six ketamine infusions.**

| Subcortical regions | Total | |  | RES | |  | NRES | |
| --- | --- | --- | --- | --- | --- | --- | --- | --- |
|  | t | *P* |  | t | *P* |  | t | *P* |
| **Left** |  |  |  |  |  |  |  |  |
| Thalamus | 0.536 | 0.595 |  | 0.115 | 0.909 |  | 1.393 | 0.183 |
| Caudate | 0.096 | 0.924 |  | 0.682 | 0.501 |  | -1.918 | 0.073 |
| Putamen | 1.310 | 0.197 |  | 1.265 | 0.217 |  | 0.342 | 0.737 |
| Pallidum | -0.581 | 0.564 |  | 0.521 | 0.607 |  | -2.287 | 0.036 |
| Hippocampus | 1.369 | 0.178 |  | 1.040 | 0.308 |  | 0.876 | 0.394 |
| Amygdala | 3.438 | 0.001* |  | 3.310 | 0.003* |  | 1.339 | 0.199 |
| **Right** |  |  |  |  |  |  |  |  |
| Thalamus | 1.311 | 0.197 |  | 1.111 | 0.277 |  | 0.872 | 0.396 |
| Caudate | 1.983 | 0.054 |  | 1.808 | 0.082 |  | 0.822 | 0.423 |
| Putamen | 0.395 | 0.695 |  | 0.732 | 0.471 |  | -1.202 | 0.247 |
| Pallidum | 0.137 | 0.891 |  | 0.836 | 0.411 |  | -1.032 | 0.318 |
| Hippocampus | 2.983 | 0.005* |  | 2.596 | 0.015 |  | 1.459 | 0.164 |
| Amygdala | 2.308 | 0.026 |  | 2.611 | 0.015 |  | 0.291 | 0.775 |

MDD, major depressive disorder; RES, responders; NRES, non-responders;

**P*-value significant after the Bonferroni correction.

**Supplementary table 4. Results of a post-hoc paired t-test for each hippocampal subfield between before and after six ketamine infusions**

| Subfields | Total | |  | RES | |  | NRES | |
| --- | --- | --- | --- | --- | --- | --- | --- | --- |
|  | t | *P* |  | t | *P* |  | t | *P* |
| **Left** |  |  |  |  |  |  |  |  |
| CA1 head | -0.563 | 0.576 |  | -0.844 | 0.407 |  | 0.212 | 0.835 |
| CA1 body | 3.066 | 0.004 |  | 3.388 | 0.002* |  | 0.669 | 0.513 |
| CA3 head | 0.539 | 0.593 |  | 0.438 | 0.665 |  | 0.307 | 0.763 |
| CA3 body | 0.399 | 0.692 |  | 0.578 | 0.568 |  | -0.017 | 0.987 |
| CA4 head | 0.303 | 0.763 |  | -0.126 | 0.901 |  | 0.688 | 0.501 |
| CA4 body | 3.989 | <0.001* |  | 5.201 | <0.001* |  | 0.158 | 0.877 |
| Fimbria | 1.780 | 0.082 |  | 1.169 | 0.253 |  | 1.423 | 0.174 |
| GC-ML-DG head | 0.324 | 0.748 |  | -0.155 | 0.878 |  | 0.754 | 0.462 |
| GC-ML-DG body | 4.401 | <0.001* |  | 5.518 | <0.001* |  | 0.284 | 0.780 |
| HATA | -0.778 | 0.441 |  | -0.973 | 0.340 |  | 0.210 | 0.836 |
| Fissure | 0.693 | 0.492 |  | -1.018 | 0.318 |  | 2.407 | 0.029 |
| Tail | 1.708 | 0.095 |  | 1.544 | 0.135 |  | 0.734 | 0.474 |
| ML head | 1.921 | 0.061 |  | 2.064 | 0.049 |  | 0.702 | 0.493 |
| ML body | 0.950 | 0.348 |  | 0.212 | 0.834 |  | 1.073 | 0.299 |
| Para | 1.474 | 0.148 |  | 0.176 | 0.861 |  | 2.420 | 0.028 |
| Pre head | 0.553 | 0.583 |  | 0.493 | 0.626 |  | 0.257 | 0.800 |
| Pre body | 1.548 | 0.129 |  | 0.667 | 0.511 |  | 1.484 | 0.157 |
| Sub head | 2.212 | 0.032 |  | 2.640 | 0.014 |  | -0.316 | 0.756 |
| Sub body | 2.473 | 0.017 |  | 0.634 | 0.532 |  | 5.263 | <0.001* |
| **Right** |  |  |  |  |  |  |  |  |
| CA1 head | 0.403 | 0.689 |  | 0.292 | 0.773 |  | 0.360 | 0.723 |
| CA1 body | 0.811 | 0.422 |  | 0.822 | 0.419 |  | 0.271 | 0.790 |
| CA3 head | 1.751 | 0.087 |  | 1.917 | 0.066 |  | 0.352 | 0.729 |
| CA3 body | 0.377 | 0.708 |  | 0.382 | 0.706 |  | 0.152 | 0.881 |
| CA4 head | 3.677 | 0.001* |  | 2.714 | 0.012 |  | 2.470 | 0.025 |
| CA4 body | 1.931 | 0.060 |  | 1.310 | 0.202 |  | 1.427 | 0.173 |
| Fimbria | 1.689 | 0.098 |  | 1.626 | 0.116 |  | 0.530 | 0.603 |
| GC-ML-DG head | 1.471 | 0.149 |  | 1.704 | 0.100 |  | 0.179 | 0.861 |
| GC-ML-DG body | 3.008 | 0.004 |  | 5.066 | <0.001* |  | -0.162 | 0.873 |
| HATA | 1.153 | 0.255 |  | 2.023 | 0.054 |  | -0.462 | 0.651 |
| Fissure | -0.029 | 0.977 |  | 0.513 | 0.612 |  | -0.539 | 0.597 |
| Tail | -0.604 | 0.549 |  | 0.485 | 0.632 |  | -1.483 | 0.157 |
| ML head | 3.500 | 0.001* |  | 3.700 | 0.001* |  | 1.320 | 0.205 |
| ML body | 1.702 | 0.096 |  | 1.446 | 0.160 |  | 0.919 | 0.372 |
| Para | 1.121 | 0.269 |  | 1.180 | 0.249 |  | 0.313 | 0.759 |
| Pre head | 1.050 | 0.300 |  | 0.619 | 0.541 |  | 0.888 | 0.387 |
| Pre body | 1.739 | 0.089 |  | 1.502 | 0.145 |  | 0.901 | 0.381 |
| Sub head | 0.361 | 0.720 |  | 0.140 | 0.890 |  | 0.386 | 0.705 |
| Sub body | 1.485 | 0.145 |  | 1.850 | 0.076 |  | -0.274 | 0.787 |

MDD, major depressive disorder; RES, responders; NRES, non-responders. GCL, granule cell layer; ML, molecular layer; CA, cornu ammonis; DG, dentate gyrus; HATA, hippocampal-amygdaloid transition area; Para, parasubiculum; Pre, presubiculum; Sub, subiculum.

**P*-value significant after the Bonferroni correction.

**Supplementary table 5. Results of partial correlation analyses between the change of MADRS scores and the volumes of subcortical regions pre-treatment and their change after ketamine treatment**

| Subcortical regions | Baseline volume | |  | Change in volume | |
| --- | --- | --- | --- | --- | --- |
|  | r | *P* |  | r | *P* |
| **Left** |  |  |  |  |  |
| Thalamus | 0.357 | 0.022 |  | -0.249 | 0.116 |
| Caudate | 0.079 | 0.623 |  | -0.180 | 0.260 |
| Putamen | 0.176 | 0.271 |  | -0.283 | 0.073 |
| Pallidum | 0.341 | 0.029 |  | -0.141 | 0.380 |
| Hippocampus | 0.059 | 0.715 |  | -0.221 | 0.164 |
| Amygdala | -0.213 | 0.182 |  | -0.452 | 0.003* |
| **Right** |  |  |  |  |  |
| Thalamus | 0.501 | 0.001* |  | -0.358 | 0.021 |
| Caudate | 0.124 | 0.440 |  | -0.148 | 0.357 |
| Putamen | 0.160 | 0.317 |  | -0.194 | 0.224 |
| Pallidum | 0.187 | 0.242 |  | -0.270 | 0.087 |
| Hippocampus | -0.120 | 0.455 |  | -0.346 | 0.027 |
| Amygdala | -0.014 | 0.932 |  | -0.392 | 0.011 |

MADRS, Montgomery-Asberg Scale.

**P*-value significant after the Bonferroni correction.

**Supplementary table 6. Results of partial correlation analyses between the change of MADRS scores and the volumes of hippocampal subfields pre-treatment and their change after ketamine treatment**

| Subfields | Baseline volume | |  | Change in volume | |
| --- | --- | --- | --- | --- | --- |
|  | r | *P* |  | r | *P* |
| **Left** |  |  |  |  |  |
| CA1 head | 0.277 | 0.079 |  | 0.212 | 0.183 |
| CA1 body | 0.086 | 0.592 |  | -0.301 | 0.056 |
| CA3 head | 0.283 | 0.073 |  | 0.034 | 0.832 |
| CA3 body | 0.019 | 0.906 |  | 0.092 | 0.568 |
| CA4 head | 0.147 | 0.359 |  | -0.100 | 0.533 |
| CA4 body | -0.023 | 0.889 |  | -0.537 | <0.001* |
| Fimbria | 0.325 | 0.038 |  | 0.197 | 0.217 |
| GC-ML-DG head | 0.215 | 0.178 |  | -0.075 | 0.643 |
| GC-ML-DG body | -0.016 | 0.923 |  | -0.301 | 0.056 |
| HATA | 0.147 | 0.360 |  | -0.170 | 0.287 |
| Fissure | 0.276 | 0.080 |  | 0.407 | 0.008 |
| Tail | 0.132 | 0.411 |  | 0.059 | 0.716 |
| ML head | 0.250 | 0.115 |  | 0.061 | 0.706 |
| ML body | -0.002 | 0.991 |  | 0.095 | 0.553 |
| Para | 0.211 | 0.186 |  | 0.312 | 0.047 |
| Pre head | -0.025 | 0.877 |  | -0.169 | 0.290 |
| Pre body | 0.025 | 0.878 |  | -0.024 | 0.880 |
| Sub head | 0.471 | 0.002* |  | -0.178 | 0.265 |
| Sub body | 0.137 | 0.392 |  | 0.257 | 0.105 |
| **Right** |  |  |  |  |  |
| CA1 head | 0.312 | 0.047 |  | -0.092 | 0.569 |
| CA1 body | 0.114 | 0.478 |  | 0.049 | 0.759 |
| CA3 head | 0.321 | 0.041 |  | -0.067 | 0.679 |
| CA3 body | 0.250 | 0.114 |  | 0.094 | 0.560 |
| CA4 head | 0.296 | 0.060 |  | 0.047 | 0.772 |
| CA4 body | 0.327 | 0.037 |  | 0.045 | 0.780 |
| Fimbria | 0.185 | 0.246 |  | -0.013 | 0.936 |
| GC-ML-DG head | -0.364 | 0.019 |  | -0.099 | 0.540 |
| GC-ML-DG body | 0.132 | 0.410 |  | -0.141 | 0.380 |
| HATA | 0.218 | 0.171 |  | -0.089 | 0.578 |
| Fissure | 0.174 | 0.277 |  | -0.336 | 0.032 |
| Tail | 0.186 | 0.245 |  | -0.055 | 0.731 |
| ML head | 0.284 | 0.071 |  | -0.067 | 0.677 |
| ML body | 0.287 | 0.069 |  | -0.016 | 0.920 |
| Para | 0.158 | 0.323 |  | -0.054 | 0.735 |
| Pre head | 0.109 | 0.498 |  | 0.103 | 0.521 |
| Pre body | 0.076 | 0.638 |  | 0.123 | 0.445 |
| Sub head | -0.045 | 0.781 |  | -0.164 | 0.306 |
| Sub body | -0.013 | 0.934 |  | -0.209 | 0.191 |

MADRS, Montgomery-Asberg Scale; GCL, granule cell layer; ML, molecular layer; CA, cornu ammonis; DG, dentate gyrus; HATA, hippocampal-amygdaloid transition area; Para, parasubiculum; Pre, presubiculum; Sub, subiculum.

**P*-value significant after the Bonferroni correction.
